# Factors Affecting Time to First Treatment in Early-Stage Chronic Lymphocytic Leukemia: A Single-Center Retrospective Cohort Study

**DOI:** 10.3390/diagnostics16213532

**Published:** 2026-10-28

**Authors:** Meryem Şener, Mehmet Ali Uçar, Emel Gürkan

**Affiliations:** Department of Hematology, Faculty of Medicine, Çukurova University, 01330 Adana, Türkiye

**Keywords:** chronic lymphocytic leukemia, time to first treatment, Binet stage, Rai stage, absolute lymphocyte count, neutrophil-to-lymphocyte ratio, real-world data

## Abstract

**Background/Objectives:** Most patients with chronic lymphocytic leukemia (CLL) are diagnosed at an early stage, but the time to first treatment (TTFT) varies widely. We aimed to identify clinical and routine laboratory factors associated with TTFT. **Methods:** We retrospectively analyzed 103 patients with Rai stage I–II CLL followed at a tertiary center between 2005 and 2025. TTFT was the primary endpoint, and overall survival (OS) the secondary endpoint; Kaplan–Meier, competing-risk, and Cox regression analyses were performed. **Results:** Median age was 71 years; 78 patients (75.7%) had Binet stage B disease. During a median follow-up of 125.8 months, 66 patients (64.1%) received treatment (median TTFT, 38.1 months). Only 1 of 25 Binet stage A patients required treatment, versus 65 of 78 Binet stage B patients (median TTFT, 20.2 months; *p* < 0.001). In multivariable analysis, Binet stage B (hazard ratio [HR] 34.8, 95% confidence interval [CI] 4.8–252.4) and a higher absolute lymphocyte count (ALC; HR 1.33 per doubling, 95% CI 1.10–1.61) were independently associated with a shorter TTFT, whereas age, sex, lactate dehydrogenase, and β2-microglobulin were not. The neutrophil-to-lymphocyte ratio added no information beyond ALC. No factor was independently associated with OS. **Conclusions:** In this tertiary referral cohort of Rai stage I–II CLL without Rai stage 0 patients, Binet stage and lymphocyte count, available from a physical examination and a complete blood count, identified patients likely to need treatment soon and may guide follow-up when molecular markers are unavailable. These findings should not be extrapolated to all patients with asymptomatic early-stage CLL.

## 1. Introduction

Chronic lymphocytic leukemia (CLL) is the most common leukemia in adults in Western countries and occurs predominantly in older individuals [1]. Most patients are diagnosed at an early, asymptomatic stage, frequently after lymphocytosis is detected incidentally on a complete blood count performed for another reason [1,2]. The clinical course is highly heterogeneous: some patients remain stable for many years without ever requiring therapy, whereas others progress rapidly and need treatment shortly after diagnosis [1].

For patients with early-stage, asymptomatic disease, a watch-and-wait strategy remains the standard of care. Early treatment with chlorambucil did not improve survival in randomized trials and their meta-analysis [3,4]. More recently, neither early chemoimmunotherapy with fludarabine, cyclophosphamide, and rituximab (FCR) in high-risk Binet stage A disease (CLL7 trial) [5] nor early ibrutinib (CLL12 trial) [6] translated into an overall survival benefit, and treatment is therefore recommended only when the International Workshop on CLL (iwCLL) criteria for active disease are met [2,7]. In this setting, time to first treatment (TTFT) is a clinically meaningful endpoint: it reflects the pace of disease progression and determines how closely patients need to be followed and how they are counseled at diagnosis.

The Rai and Binet staging systems, which rely only on physical examination and a complete blood count, remain the cornerstone of clinical assessment [8,9], but they were developed to predict survival and discriminate less well within the lower stages, where most newly diagnosed patients are found. Unmutated immunoglobulin heavy-chain variable region (IGHV) genes [10,11] and cytogenetic abnormalities detected by fluorescence in situ hybridization (FISH), particularly del(17p) and del(11q) [12], identify patients with a more aggressive course, and serum β2-microglobulin (B2M) adds prognostic information [13]. These factors have been integrated into the CLL international prognostic index (CLL-IPI) [13,14], which also predicts TTFT in early-stage disease [15], and into the international prognostic score for asymptomatic early-stage CLL (IPS-E), developed specifically to predict TTFT [16].

In routine practice, however, IGHV and *TP53* mutational analyses are unavailable in many centers, and FISH is often requested only when treatment is being considered, so prognostic assessment at diagnosis relies largely on clinical stage and the complete blood count. An absolute lymphocyte count (ALC) > 15 × 10^9^/L is one of the three components of the IPS-E [16]. The neutrophil-to-lymphocyte ratio (NLR), an inexpensive marker with prognostic value in many solid tumors [17], has also attracted interest because neutrophils are increasingly recognized in the CLL microenvironment [18]; however, data on NLR and treatment need in CLL are scarce and inconsistent [19]. The prognostic value of B2M may be influenced by renal function, which is relevant in elderly patients with comorbidities [20]. Because early-stage CLL is frequently detected incidentally on a routine blood count, simple prognostic markers available at diagnosis that identify patients likely to need treatment soon are of practical importance.

Real-world data on TTFT and its determinants in patients managed outside clinical trials, in whom molecular markers are often unavailable, remain limited. Therefore, the aim of this study was to evaluate TTFT and to identify the clinical and routine laboratory factors associated with TTFT in patients with Rai stage I–II CLL followed at a single tertiary center. As a secondary aim, we evaluated overall survival (OS) and its associated factors in the same cohort.

## 2. Materials and Methods

### 2.1. Study Design and Patient Population

This single-center, retrospective cohort study was conducted at the Department of Hematology, Faculty of Medicine, Çukurova University, Adana, Türkiye. The medical records of all 163 patients diagnosed with CLL and followed at our center between 2005 and 2025 (the period covering the dates of diagnosis and follow-up of the patients) were screened. This period refers to the clinical course of the patients and not to the period of data collection; all data were retrieved retrospectively from the medical records between October and December 2025, after ethics approval had been obtained (Section 2.2). CLL was diagnosed according to the iwCLL criteria [2]. Disease stage at diagnosis was determined according to both the Rai and Binet staging systems [8,9] from the physical examination and complete blood count documented in the medical records at diagnosis.

Patients with Rai stage I–II disease at diagnosis were eligible. Patients with Rai stage III–IV or Binet stage C disease (*n* = 49) and those whose stage at diagnosis could not be determined from the medical records (*n* = 11) were excluded; no patient was recorded as having Rai stage 0 disease. The final study population therefore comprised 103 patients with Rai stage I–II disease, of whom 25 had Binet stage A and 78 Binet stage B disease. Because Rai stage II and Binet stage B disease are often classified as intermediate rather than early stage, the cohort is described by its Rai stage throughout.

### 2.2. Ethics

The study was approved by the Clinical Research Ethics Committee of Çukurova University Faculty of Medicine (meeting no. 146, decision no. 32, dated 26 July 2024) and was conducted in accordance with the principles of the Declaration of Helsinki. Owing to the retrospective design of the study, the requirement for written informed consent was waived by the ethics committee. Ethics approval was obtained before any data were collected; the medical records were reviewed and the study data were extracted between October and December 2025, after the date of approval.

### 2.3. Data Collection

Demographic, clinical, and laboratory data were retrieved from the hospital records: age, sex, Rai and Binet stage at diagnosis, comorbidities, complete blood count at diagnosis (hemoglobin, platelet count, and absolute lymphocyte and neutrophil counts), serum lactate dehydrogenase (LDH) and B2M levels, FISH results, indication for, date, regimen, and response of first-line treatment, date of last follow-up, and date of death. NLR was calculated as the absolute neutrophil count divided by the ALC.

The FISH panel included probes for del(17p)/*TP53*, del(11q22), del(13q14), 13q34, and trisomy 12. Because FISH testing was not routinely performed at diagnosis and was requested at the discretion of the treating physician, usually once a decision to initiate treatment had been made, FISH results are reported descriptively, and FISH status was not used as a prognostic covariate.

### 2.4. Treatment and Response Assessment

Patients were managed with a watch-and-wait approach until the development of active disease. The decision to start treatment was made by the treating hematologist on the basis of the iwCLL criteria for active disease [2], most commonly constitutional (B) symptoms, progressive or bulky lymphadenopathy, and/or progressive splenomegaly or hepatosplenomegaly. Response to first-line treatment was categorized as complete response (CR), partial response (PR), stable disease (SD), or progressive disease (PD) according to the iwCLL response criteria [2].

### 2.5. Endpoints

The primary endpoint was TTFT, defined as the time from the date of diagnosis to the initiation of first-line therapy; patients who had not received treatment were censored at the date of last follow-up. The secondary endpoint was OS, defined as the time from diagnosis to death from any cause; surviving patients were censored at the date of last follow-up. Treatment-free survival (TFS), defined as the time from diagnosis to first treatment or death from any cause, whichever occurred first, was analyzed as a supplementary endpoint.

### 2.6. Statistical Analysis

Categorical variables were summarized as numbers and percentages, and continuous variables as mean ± standard deviation or median (interquartile range [IQR] or range). For variables with missing data, percentages were calculated among patients with available data. Median follow-up was estimated with the reverse Kaplan–Meier method.

TTFT, TFS, and OS were estimated using the Kaplan–Meier method, and subgroups were compared using the log-rank test; 1-, 2-, and 5-year rates were calculated. Because death before treatment precludes subsequent treatment, the cumulative incidence of first treatment was also estimated with the Aalen–Johansen estimator, treating death without treatment as a competing event. ALC and B2M were dichotomized at externally established thresholds (ALC > 15 × 10^9^/L, as in the IPS-E [16]; B2M ≥ 3.5 mg/L, as in the CLL-IPI [13]) rather than at data-derived cut-offs.

Factors associated with TTFT were evaluated using univariable and multivariable Cox proportional hazards regression (cause-specific hazards), and results were expressed as hazard ratios (HRs) with 95% confidence intervals (CIs). Age and LDH were analyzed as continuous variables; ALC and NLR, which were right-skewed, were log_2_-transformed, so that their HRs correspond to a doubling of the value. The multivariable model included age and sex as prespecified covariates, together with Binet stage and log_2_ ALC; Rai and Binet stages were not entered into the same model because of their overlap. Sensitivity analyses replaced Binet with Rai stage, replaced continuous ALC with ALC > 15 × 10^9^/L, excluded patients treated within 3 months of diagnosis, and added log_2_ NLR to the model. Factors associated with OS were evaluated with univariable and multivariable Cox regression, including age group (<65 vs. ≥65 years), sex, Binet stage, and ALC > 15 × 10^9^/L; B2M was analyzed in univariable models only because it was missing in one-fifth of patients. Variables measured after diagnosis, such as response to treatment, were not included in OS models. The proportional hazards assumption was checked using Schoenfeld residuals, and analyses were performed on a complete-case basis.

Statistical analyses were performed using IBM SPSS Statistics for Windows, version 25.0 (IBM Corp., Armonk, NY, USA) and Python (version 3.13; Python Software Foundation, Wilmington, DE, USA), which was also used to generate the figure. A two-sided *p*-value < 0.05 was considered statistically significant. The study was reported in accordance with the Strengthening the Reporting of Observational Studies in Epidemiology (STROBE) guidelines.

## 3. Results

### 3.1. Patient Characteristics

Baseline characteristics of the 103 patients are summarized in Table 1. The mean age at diagnosis was 69.6 ± 12.1 years (median, 71 years; range, 40–91), and 64 patients (62.1%) were aged 65 years or older. Fifty-seven patients (55.3%) were male. At diagnosis, 21 patients (20.4%) had Rai stage I and 82 (79.6%) had Rai stage II disease; according to the Binet system, 25 patients (24.3%) were stage A and 78 (75.7%) were stage B. The median ALC was 19.4 × 10^9^/L (IQR, 11.1–34.2), and 64 of 102 patients (62.7%) had an ALC > 15 × 10^9^/L; the median NLR was 0.28 (IQR, 0.17–0.54). FISH testing had been performed in 49 patients (47.6%); of these, 20 had at least one abnormality, most commonly del(13q14) (*n* = 10), trisomy 12 (*n* = 8), del(17p)/*TP53* (*n* = 4), and del(11q) (*n* = 2). At least one comorbidity was recorded in 63 patients (61.2%).

The median follow-up was 125.8 months (95% CI, 84.3–149.7). During follow-up, 66 patients (64.1%) received first-line therapy, most commonly bendamustine with or without rituximab (*n* = 17), FCR (*n* = 16), and cyclophosphamide, vincristine, and prednisone with or without rituximab (CVP/R-CVP; *n* = 14) (Table 1); 19 of the 66 treated patients (28.8%) started treatment within 3 months of diagnosis. Among the 65 patients evaluable for response, the overall response rate was 76.9% (CR 7.7%, PR 69.2%). By the end of follow-up, 50 patients (48.5%) had died, 17 of them without having received treatment (12 with Binet stage A disease; median age, 80 years).

All 19 patients who started treatment within 3 months of diagnosis had Rai stage II/Binet stage B disease and met the iwCLL criteria for active disease at presentation: widespread, bulky, or conglomerate lymphadenopathy (*n* = 13), hepatosplenomegaly or splenomegaly (*n* = 11), and/or B symptoms (*n* = 8); none were treated for cytopenia. Compared with patients treated later (*n* = 47), they had a similar median age (68 vs. 69 years) and ALC (28.0 vs. 19.8 × 10^9^/L; *p* = 0.244) but a higher LDH (262 vs. 188 U/L; *p* = 0.031). FISH had been performed in 14 of these patients, and three of the four patients with del(17p) in the cohort belonged to this group.

### 3.2. Time to First Treatment (Primary Endpoint)

The median TTFT for the entire cohort was 38.1 months (95% CI, 20.2–54.3), and the estimated proportions of patients remaining treatment-free at 1, 2, and 5 years were 71.3%, 58.9%, and 37.8%, respectively (Table 2). With death as a competing event, the cumulative incidence of treatment was 28.7%, 41.1%, and 60.8% at 1, 2, and 5 years.

**Figure 1:**
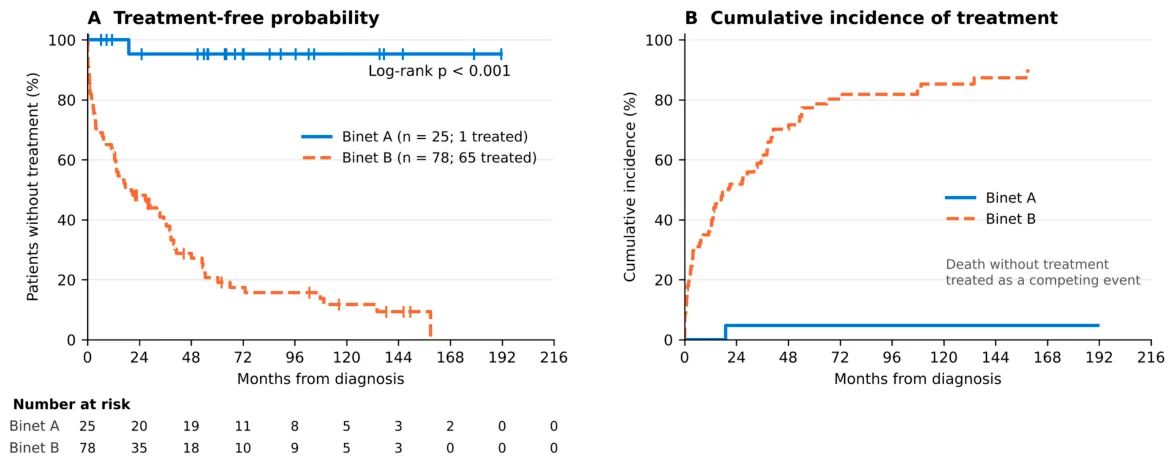
Time to first treatment according to Binet stage in 103 patients with Rai stage I–II chronic lymphocytic leukemia. (**A**) Kaplan–Meier estimates of the proportion of patients remaining treatment-free (log-rank *p* < 0.001); tick marks indicate censored observations. (**B**) Cumulative incidence of first treatment estimated with the Aalen–Johansen method, treating death without treatment as a competing event.

TTFT differed markedly according to disease stage (Table 2, Figure 1). Only 1 of the 21 patients with Rai stage I disease required treatment during follow-up, whereas patients with Rai stage II disease had a median TTFT of 21.1 months (95% CI, 13.1–36.5) (log-rank *p* < 0.001). Similarly, 1 of 25 patients with Binet stage A disease required treatment, compared with 65 of 78 patients with Binet stage B disease (median TTFT, 20.2 months [95% CI, 12.5–36.5]; log-rank *p* < 0.001). The 5-year cumulative incidence of treatment was 4.8% in Binet stage A and 77.3% in Binet stage B. TFS showed the same pattern (median, 102.3 vs. 20.2 months; *p* < 0.001), with the events in Binet stage A being mostly deaths without treatment.

Patients with an ALC > 15 × 10^9^/L had a numerically shorter TTFT than those with a lower ALC (median, 32.2 vs. 54.5 months; *p* = 0.101). TTFT did not differ significantly by age group (median, 17.6 months in patients aged <65 years vs. 41.2 months in those aged ≥65 years; *p* = 0.324), sex (*p* = 0.537), or B2M (*p* = 0.163). Patients in whom FISH testing had been performed had a shorter TTFT than those without FISH testing (median, 14.9 vs. 53.4 months; *p* = 0.018), consistent with testing being requested around the time of treatment decisions.

B2M was more frequently missing in patients with Binet stage A disease and in those who were never treated (patients without vs. with B2M: Binet stage B, 52.4% vs. 81.7%, *p* = 0.009; treated during follow-up, 28.6% vs. 73.2%, *p* < 0.001), indicating that B2M was preferentially measured in patients with more active disease. Serum creatinine at diagnosis was unavailable; excluding the four patients with documented chronic kidney disease did not change the association of B2M ≥ 3.5 mg/L with TTFT (HR 1.55, 95% CI 0.91–2.63; *p* = 0.105) or OS (HR 1.51, 95% CI 0.77–2.95; *p* = 0.226).

The results of the Cox regression analyses for TTFT are shown in Table 3. In univariable analysis, Rai stage II (HR 31.2; 95% CI, 4.3–225.8) and Binet stage B (HR 39.1; 95% CI, 5.4–283.0) were associated with a markedly higher hazard of treatment, as was a higher ALC (HR 1.30 per doubling; 95% CI, 1.09–1.57; *p* = 0.005). In contrast, a higher NLR was associated with a lower hazard of treatment (HR 0.82 per doubling; 95% CI, 0.68–0.99; *p* = 0.041), whereas age, sex, LDH, and B2M were not significantly associated with TTFT.

In the multivariable model (102 patients, 65 treatment events), Binet stage B (adjusted HR 34.8; 95% CI, 4.8–252.4; *p* < 0.001) and a higher ALC (adjusted HR 1.33 per doubling; 95% CI, 1.10–1.61; *p* = 0.004) were independently associated with a shorter TTFT, whereas age and sex were not. The association of ALC persisted when Rai stage replaced Binet stage (HR 1.26; 95% CI, 1.04–1.52), when ALC was dichotomized at 15 × 10^9^/L (HR 2.06; 95% CI, 1.19–3.58), and after exclusion of the 19 patients treated within 3 months of diagnosis (HR 1.32; 95% CI, 1.05–1.66). When log_2_ NLR was added to the model, neither ALC (*p* = 0.381) nor NLR (*p* = 0.521) remained significant, reflecting the strong dependence of NLR on the lymphocyte count. No violation of the proportional hazards assumption was detected.

### 3.3. Overall Survival (Secondary Endpoint)

In the overall cohort, the 2- and 5-year OS rates were 97.9% and 85.2%, respectively, and the median OS was 110.5 months (95% CI, 95.9–146.3) (Table 4). In Kaplan–Meier analysis, OS was shorter in patients aged ≥65 years (5-year OS, 82.3% vs. 90.5%; *p* = 0.043), in men (79.8% vs. 92.1%; *p* = 0.036), and in patients with ALC > 15 × 10^9^/L (77.5% vs. 97.1%; *p* = 0.043). OS did not differ according to Rai stage (*p* = 0.828), Binet stage (*p* = 0.377), B2M (*p* = 0.233), or FISH testing status (*p* = 0.422).

In multivariable Cox regression including age group, sex, Binet stage, and ALC, none of these factors was independently associated with OS; the HRs were 1.60 (95% CI, 0.73–3.48) for age ≥ 65 years, 0.57 (95% CI, 0.32–1.04) for female sex, and 1.55 (95% CI, 0.80–3.00) for ALC > 15 × 10^9^/L (Table 5).

## 4. Discussion

In this real-world cohort of 103 patients with Rai stage I–II CLL, nearly two-thirds required treatment during a median follow-up of more than 10 years, and the median TTFT was 38.1 months. Binet stage was the strongest determinant of treatment need: only 1 of 25 patients with Binet stage A disease was treated, whereas the median TTFT was 20.2 months in those with Binet stage B disease. A higher ALC was independently associated with a shorter TTFT, whereas the NLR was inversely associated with treatment need and added no information beyond ALC. No factor was independently associated with OS.

Because early treatment has not improved survival in patients with early-stage CLL [3,4,5,6], treatment is reserved for active disease according to the iwCLL criteria [2,7]. The main clinical task at diagnosis is therefore to identify patients likely to need treatment soon, so that follow-up intensity and counseling can be adjusted.

The median TTFT in our cohort was shorter than expected for an unselected early-stage population, most likely because three-quarters of patients had Binet stage B disease and no patient with Rai stage 0 disease was included. Our cohort therefore represents patients with early-to-intermediate-stage disease referred to a tertiary center rather than the broader population of incidentally diagnosed CLL. Although the Rai and Binet systems were developed to predict survival [8,9], our results show that, within Rai stage I–II disease, the distinction between Binet stage A and B strongly affects the need for treatment: about half of the patients with Binet stage B disease required treatment within 2 years, supporting close follow-up in this group, whereas longer intervals seem reasonable for Binet stage A disease. The very low treatment rate in Binet stage A should nevertheless be interpreted with caution: the group was small, the stage HRs had very wide confidence intervals, and most events in this group were deaths without treatment in elderly patients, so competing mortality probably limited the opportunity for progression to become clinically relevant.

The independent association between ALC and TTFT is biologically plausible because the lymphocyte count in CLL largely reflects the circulating tumor burden. An ALC > 15 × 10^9^/L is a component of the IPS-E [16], and our findings support its use when molecular markers are unavailable: in our cohort, each doubling of the ALC was associated with an approximately one-third higher hazard of treatment, and the association remained after adjustment for stage and in several sensitivity analyses. ALC at a single time point does not capture lymphocyte doubling time, which is an iwCLL criterion for treatment [2] and could not be assessed reliably in our retrospective data.

Contrary to findings in solid tumors [17], a higher NLR was associated with a lower, not higher, hazard of treatment. In CLL, the denominator of the NLR is dominated by the malignant clone, so a low NLR mainly signals a high lymphocyte count; accordingly, NLR lost its association when ALC was included in the model. This is consistent with the report of Levy et al., in which neutrophilia without monocytosis at diagnosis was associated with a more favorable prognosis and a longer time to treatment [19], although neutrophils may also contribute to disease-related immune dysfunction [18]. These observations suggest that NLR should not be interpreted in CLL as it is in solid tumors and that it offers no advantage over the lymphocyte count itself.

Cytogenetic abnormalities detected by FISH have well-established prognostic value in CLL, with del(17p) and del(11q) associated with an unfavorable course and isolated del(13q) with a favorable course [12]. In our cohort, however, FISH was usually requested once a decision to initiate treatment had been made, so testing status reflected the timing of treatment rather than disease biology, which explains the shorter TTFT in patients who underwent testing; FISH status was therefore not used as a prognostic covariate. Only 20 of the 49 tested patients had an abnormality, and del(17p)/*TP53* was detected in only 4, which precluded any meaningful analysis of individual abnormalities. These observations underline the need for FISH, and ideally *TP53* and IGHV mutational analysis, to be performed at baseline in future prospective cohorts.

B2M is an established prognostic marker in CLL and is a component of the CLL-IPI, with a threshold of 3.5 mg/L [13], as well as of other prognostic indices [14]; the CLL-IPI has also been shown to predict TTFT in early-stage CLL [15]. In our cohort, B2M was not significantly associated with either TTFT or OS. Serum B2M is strongly influenced by renal function, and adjustment for glomerular filtration rate has been shown to improve its ability to predict treatment-free survival [20]; this is particularly relevant in our elderly cohort with a high prevalence of comorbidities, in which renal function was not taken into account. In addition, B2M was missing in approximately one-fifth of patients, which reduced statistical power.

Age was not associated with TTFT, and the median TTFT was numerically longer in older patients, which may reflect a more conservative approach to treatment in older patients with comorbidities and the competing risk of death. Older age, male sex, and a higher ALC were associated with shorter OS in univariable analysis, but none of these associations was independent. The association with age is expected because OS in this elderly cohort reflects all-cause mortality; age was therefore included in the multivariable model as an adjustment variable rather than as a candidate prognostic factor. As the cause of death was unavailable, CLL-related and non-CLL-related deaths could not be distinguished, and the OS findings should not be interpreted as CLL-specific survival.

The IPS-E, based on unmutated IGHV, an ALC > 15 × 10^9^/L, and palpable lymph nodes [16], and the CLL-IPI [13,15] require molecular information that was unavailable in our cohort, as in many real-world centers. Our results suggest that, in such settings, clinical stage and the lymphocyte count still provide useful, albeit limited, risk stratification. This is relevant because early-stage CLL is often detected on a routine blood count and may be followed outside specialized hematology clinics, where these simple parameters are always available.

Some limitations should be considered when interpreting our findings. First, this was a retrospective, single-center study at a tertiary referral center, with potential selection bias and missing data; stage at diagnosis was assigned retrospectively from the medical records. Second, the sample size was modest; only one treatment event occurred in each of the Rai stage I and Binet stage A groups, so the stage HRs were imprecise. Third, the cohort included no patients with Rai stage 0 disease and was dominated by Rai stage II/Binet stage B disease, limiting generalizability; 19 patients started treatment within 3 months of diagnosis, although excluding them did not change the results. Fourth, IGHV and *TP53* mutational analyses were unavailable, so the CLL-IPI and IPS-E could not be calculated; FISH was not performed systematically at diagnosis; and lymphocyte doubling time could not be assessed. Fifth, B2M was missing in approximately 20% of patients and was not adjusted for renal function. Sixth, treatment standards changed considerably over the long study period (2005–2025); most first-line regimens were chemotherapy-based, treatment decisions were made by the treating physicians, and response was assessed retrospectively. Seventh, the cause of death was unavailable. Finally, multiple subgroup comparisons were not adjusted for multiplicity.

## 5. Conclusions

In this real-world cohort of patients with Rai stage I–II CLL, Binet stage at diagnosis was the strongest determinant of the need for treatment, and a higher lymphocyte count was independently associated with earlier treatment. The NLR was inversely related to treatment need and added no information beyond the lymphocyte count. Both stage and lymphocyte count are available from a physical examination and a complete blood count and can guide follow-up intensity where molecular markers are unavailable; prospective studies incorporating baseline FISH, IGHV, and *TP53* analyses are needed to refine risk stratification. No factor was independently associated with OS, which reflected all-cause mortality and could not be separated into CLL-related and non-CLL-related deaths. Because B2M was available in only a subset of patients, was preferentially measured in those with more active disease, and could not be adjusted for renal function, the B2M findings may be affected by informative bias and should not be generalized to the entire study population. Because the cohort was a tertiary referral population that included no patients with Rai stage 0 disease and was dominated by Binet stage B disease, these findings should not be extrapolated to the full spectrum of patients with asymptomatic early-stage CLL.

## Figures and Tables

**Table 1:** Baseline demographic and clinical characteristics of the patients (*n* = 103).

Characteristic	Value
**Age, years**	
Mean ± standard deviation	69.6 ± 12.1
Median (range)	71 (40–91)
<65	39 (37.9)
≥65	64 (62.1)
**Sex**	
Male	57 (55.3)
Female	46 (44.7)
**Rai stage at diagnosis**	
I	21 (20.4)
II	82 (79.6)
**Binet stage at diagnosis**	
A	25 (24.3)
B	78 (75.7)
**Laboratory values at diagnosis, median (IQR)**	
Absolute lymphocyte count, ×10^9^/L (*n* = 102)	19.4 (11.1–34.2)
Absolute lymphocyte count > 15 × 10^9^/L (*n* = 102), *n* (%)	64 (62.7)
Neutrophil-to-lymphocyte ratio (*n* = 100)	0.28 (0.17–0.54)
Hemoglobin, g/dL (*n* = 102)	13.1 (12.3–14.1)
Platelet count, ×10^9^/L (*n* = 102)	199 (160–252)
Lactate dehydrogenase, U/L (*n* = 102)	186 (157–255)
β2-microglobulin, mg/L (*n* = 82)	3.28 (2.30–4.22)
β2-microglobulin ≥ 3.5 mg/L (*n* = 82), *n* (%)	32 (39.0)
**FISH testing**	
Not performed	54 (52.4)
Performed	49 (47.6)
≥1 abnormality detected ^a^	20 (40.8)
**Comorbidity**	
Absent	40 (38.8)
Present	63 (61.2)
**First-line treatment (*n* = 66) ^b^**	
Bendamustine ± rituximab	17 (25.8)
FCR	16 (24.2)
CVP/R-CVP	14 (21.2)
Chlorambucil ± anti-CD20 antibody	8 (12.1)
Ibrutinib	5 (7.6)
Venetoclax-based	3 (4.5)
Other (R-CHOP, cyclophosphamide)	3 (4.5)
**Response to first-line treatment (*n* = 65) ^b^**	
CR	5 (7.7)
PR	45 (69.2)
SD	11 (16.9)
PD	4 (6.2)
**Vital status at last follow-up**	
Alive	53 (51.5)
Deceased	50 (48.5)
Deceased without treatment	17 (16.5)
**Median follow-up, months (95% CI) ^c^**	125.8 (84.3–149.7)

Data are presented as *n* (%) unless otherwise indicated. Variable names are shown in bold. ^a^ percentage among patients with FISH testing (*n* = 49): del(13q14) *n* = 10, trisomy 12 *n* = 8, del(17p)/*TP53 n* = 4, del(11q) *n* = 2, other *n* = 4; some patients had more than one abnormality. ^b^ percentages are calculated among patients who received first-line treatment (*n* = 66) or were evaluable for response (*n* = 65). ^c^ reverse Kaplan–Meier method. Abbreviations: IQR, interquartile range; FISH, fluorescence in situ hybridization; FCR, fludarabine, cyclophosphamide, rituximab; CVP, cyclophosphamide, vincristine, prednisone; R, rituximab; CHOP, cyclophosphamide, doxorubicin, vincristine, prednisone; CR, complete response; PR, partial response; SD, stable disease; PD, progressive disease.

**Table 2:** Time to first treatment in the overall cohort and according to clinical and laboratory subgroups (Kaplan–Meier analysis).

Variable	*n*	Events	Censored	Median TTFT, Months (95% CI)	1-Year TF (%)	2-Year TF (%)	5-Year TF (%)	Log-Rank *p*
**Overall**	103	66	37	38.1 (20.2–54.3)	71.3	58.9	37.8	—
**Age**								0.324
<65 years	39	27	12	17.6 (12.5–54.5)	68.6	43.9	31.7	
≥65 years	64	39	25	41.2 (32.2–66.0)	73.0	68.0	41.7	
**Sex**								0.537
Male	57	37	20	35.4 (11.7–66.0)	63.8	56.5	39.5	
Female	46	29	17	38.3 (16.9–107.9)	80.3	61.4	35.9	
**Rai stage**								<0.001 *
I	21	1	20	NR	100.0	100.0	94.1	
II	82	65	17	21.1 (13.1–36.5)	64.3	49.2	24.4	
**Binet stage**								<0.001 *
A	25	1	24	NR	100.0	95.2	95.2	
B	78	65	13	20.2 (12.5–36.5)	62.5	48.2	20.8	
**ALC, ×10^9^/L**								0.101
≤15	38	23	15	54.5 (29.1–158.9)	73.7	68.4	48.5	
>15	64	42	22	32.2 (14.2–48.2)	71.1	53.7	31.3	
**B2M, mg/L**								0.163
<3.5	50	33	17	38.1 (13.5–54.5)	71.8	57.5	36.0	
≥3.5	32	27	5	32.2 (3.8–41.2)	65.6	53.1	26.7	
**FISH testing ^a^**								0.018 *
Not performed	54	31	23	53.4 (32.2–109.5)	83.1	71.2	49.0	
Performed	49	35	14	14.9 (7.5–38.1)	58.4	45.4	25.7	

^a^ FISH testing was usually requested once a treatment decision had been made; this comparison is shown descriptively and does not indicate a prognostic effect. * *p* < 0.05. Variable names are shown in bold. Abbreviations: TTFT, time from diagnosis to first treatment; events, patients who received first-line treatment; TF, treatment-free (Kaplan–Meier estimate); CI, confidence interval; NR, not reached; ALC, absolute lymphocyte count (*n* = 102); B2M, β2-microglobulin (*n* = 82); FISH, fluorescence in situ hybridization.

**Table 3:** Univariable and multivariable Cox regression analyses of factors associated with time to first treatment.

Variable	Univariable HR (95% CI)	*p*	Adjusted HR (95% CI) ^a^	*p*
Age (per 1-year increase)	0.98 (0.96–1.00)	0.077	0.98 (0.96–1.00)	0.103
Female sex (ref: male)	0.86 (0.52–1.40)	0.538	1.05 (0.63–1.74)	0.858
Rai stage II (ref: I)	31.2 (4.3–225.8)	<0.001 *	— ^b^	
Binet stage B (ref: A)	39.1 (5.4–283.0)	<0.001 *	34.8 (4.8–252.4)	<0.001 *
ALC (per doubling)	1.30 (1.09–1.57)	0.005 *	1.33 (1.10–1.61)	0.004 *
ALC > 15 × 10^9^/L (ref: ≤15)	1.54 (0.92–2.58)	0.103	— ^c^	
NLR (per doubling)	0.82 (0.68–0.99)	0.041 *	— ^c^	
LDH (per 100 U/L increase)	1.11 (0.90–1.35)	0.329	—	
B2M ≥ 3.5 mg/L (ref: <3.5)	1.44 (0.86–2.40)	0.165	—	

^a^ multivariable model including age, sex, Binet stage, and ALC (*n* = 102; 65 treatment events). Univariable analyses included all patients with available data (ALC *n* = 102, NLR *n* = 100, LDH *n* = 102, B2M *n* = 82). ^b^ Rai and Binet stages were not entered into the same model; replacing Binet with Rai stage gave an adjusted HR of 25.7 (95% CI, 3.5–187.3) for Rai stage II and 1.26 (1.04–1.52) for ALC per doubling. ^c^ sensitivity analyses: ALC > 15 × 10^9^/L in place of continuous ALC, adjusted HR 2.06 (1.19–3.58), *p* = 0.010; after the exclusion of 19 patients treated within 3 months of diagnosis, ALC per doubling 1.32 (1.05–1.66), *p* = 0.018; with log_2_ NLR added, neither ALC (*p* = 0.381) nor NLR (*p* = 0.521) remained significant. * *p* < 0.05. Abbreviations: HR, hazard ratio; CI, confidence interval; ALC, absolute lymphocyte count; NLR, neutrophil-to-lymphocyte ratio; LDH, lactate dehydrogenase; B2M, β2-microglobulin.

**Table 4:** Overall survival in the overall cohort and according to clinical and laboratory subgroups (Kaplan–Meier analysis).

Variable	Deaths/*n*	2-Year OS (%)	5-Year OS (%)	Median OS, Months (95% CI)	Log-Rank *p*
**Overall**	50/103	97.9	85.2	110.5 (95.9–146.3)	—
**Age**					0.043 *
<65 years	9/39	100.0	90.5	NR	
≥65 years	41/64	96.6	82.3	102.3 (85.6–135.2)	
**Sex**					0.036 *
Male	32/57	96.2	79.8	101.7 (69.7–135.2)	
Female	18/46	100.0	92.1	149.5 (98.5–210.7)	
**Rai stage**					0.828
I	9/21	100.0	94.1	135.2 (64.2–178.9)	
II	41/82	97.4	83.2	110.5 (92.3–148.8)	
**Binet stage**					0.377
A	13/25	95.2	80.7	102.3 (64.2–137.2)	
B	37/78	98.6	86.6	122.4 (92.3–149.5)	
**ALC, ×10^9^/L**					0.043 *
≤15	14/38	100.0	97.1	146.3 (109.6–210.7)	
>15	35/64	96.5	77.5	98.5 (82.0–129.9)	
**B2M, mg/L**					0.233
<3.5	18/50	100.0	92.9	135.2 (104.9–NE)	
≥3.5	21/32	93.8	87.0	102.3 (72.2–173.8)	
**FISH testing**					0.422
Not performed	35/54	96.0	81.5	104.9 (92.3–148.8)	
Performed	15/49	100.0	89.6	110.5 (85.0–NE)	

* *p* < 0.05. Variable names are shown in bold. Abbreviations: OS, overall survival; CI, confidence interval; NR, not reached; NE, not estimable; ALC, absolute lymphocyte count (*n* = 102); B2M, β2-microglobulin (*n* = 82); FISH, fluorescence in situ hybridization.

**Table 5:** Univariable and multivariable Cox regression analyses of factors associated with overall survival.

Variable	Univariable HR (95% CI)	*p*	Adjusted HR (95% CI) ^a^	*p*
Age ≥ 65 years (ref: <65)	2.09 (1.01–4.33)	0.048 *	1.60 (0.73–3.48)	0.240
Female sex (ref: male)	0.54 (0.30–0.97)	0.039 *	0.57 (0.32–1.04)	0.068
Rai stage II (ref: I)	1.08 (0.52–2.25)	0.828	—	
Binet stage B (ref: A)	0.75 (0.40–1.42)	0.379	0.73 (0.38–1.39)	0.338
ALC > 15 × 10^9^/L (ref: ≤15)	1.89 (1.01–3.53)	0.046 *	1.55 (0.80–3.00)	0.198
B2M ≥ 3.5 mg/L (ref: <3.5) ^b^	1.48 (0.77–2.85)	0.235	—	

^a^ multivariable model including age group, sex, Binet stage, and ALC (*n* = 102; 49 deaths). ^b^ analyzed in univariable models only (*n* = 82; 39 deaths); adding B2M to the multivariable model did not change the results (adjusted HR 1.11, 95% CI 0.52–2.34). * *p* < 0.05. Abbreviations: HR, hazard ratio; CI, confidence interval; ALC, absolute lymphocyte count; B2M, β2-microglobulin.

## Data Availability

The data presented in this study are available on request from the corresponding author due to privacy and ethical restrictions.

## Abbreviations

The following abbreviations are used in this manuscript:

ALC	Absolute Lymphocyte Count
B2M	β2-Microglobulin
CI	Confidence Interval
CLL	Chronic Lymphocytic Leukemia
CLL-IPI	CLL International Prognostic Index
FISH	Fluorescence in Situ Hybridization
HR	Hazard Ratio
IGHV	Immunoglobulin Heavy-Chain Variable Region
IPS-E	International Prognostic Score for Asymptomatic Early-stage CLL
iwCLL	International Workshop on Chronic Lymphocytic Leukemia
LDH	Lactate Dehydrogenase
NLR	Neutrophil-to-Lymphocyte Ratio
OS	Overall Survival
TFS	Treatment-Free Survival
TTFT	Time to First Treatment
